# A DFT Study on the Effect of Biaxial Strain on the Electronic Properties of Graphene Doped with B, N, Al, Si, S, and Ga

**DOI:** 10.3390/ma18122791

**Published:** 2025-06-13

**Authors:** Dinara Akhmetsadyk, Daniyar Ismailov, Danatbek Murzalinov, Gulmaira Partizan, Valentina Grichshenko

**Affiliations:** 1 LLP «Institute of Ionosphere», Almaty 050020, Kazakhstan; ismailov_daniyar_v@bk.ru (D.I.); murzalinov@sci.kz (D.M.); gulmira.partizan@gmail.com (G.P.); labreab@mail.ru (V.G.); 2 National Nanotechnology Laboratory of Open Type, Al-Farabi Kazakh National University, Almaty 050040, Kazakhstan; 3 National Scientific Laboratory for Collective Use of Information and Space Technologies, Satbayev University, Almaty 050013, Kazakhstan; 4 Institute of Physics and Technology, Satbayev University, Almaty 050013, Kazakhstan

**Keywords:** graphene, strain sensors, biaxial strain, density of states, density functional theory

## Abstract

This study presents a density functional theory (DFT) investigation of the electronic response of graphene doped with various atoms (B, N, Al, Si, S, Ga) under biaxial strain. The calculations were performed using the PBE exchange–correlation functional within the generalized gradient approximation (GGA), as implemented in the DMol^3^ code. The Fermi energy was used as the primary indicator to evaluate strain sensitivity across a deformation range from −0.05 to +0.05. The results reveal a strong dependence of the electronic response on the type of dopant. Ga- and Al-doped graphene systems exhibit the most pronounced Fermi level shifts, up to 0.6 eV, indicating high sensitivity to mechanical strain. In contrast, B- and N-doped graphene show more moderate but stable and linear changes, which may be advantageous for predictable sensor behavior. These findings highlight the critical role of dopant selection in engineering strain-responsive graphene materials and support a design framework for their integration into high-performance flexible electronics and sensing applications.

## 1. Introduction

Strain and pressure sensors are critical components in various industrial sectors [1], providing essential data for monitoring structural integrity, detecting defects, and ensuring safety. They are widely used in aerospace [2,3], healthcare [4], automotive [5], robotics [6], and other applications, where precise measurements are crucial for system performance and reliability. In the space industry, strain sensors play a significant role in monitoring the condition of spacecraft materials to detect critical deformations and prevent potential structural degradation. Pressure sensors are indispensable for monitoring propulsion systems, atmospheric conditions, and life-support devices [7]. The reliability and accuracy of these sensors are fundamental to the success of space missions, where extreme environmental conditions demand robust and highly sensitive materials.

Graphene nanostructures have emerged as promising candidates for strain and pressure sensors in space applications due to their exceptional mechanical strength, high electrical conductivity, and flexibility. First isolated in 2004, graphene continues to be the subject of intense research because its characteristics make it ideal for applications in electronics, sensor systems, and new materials. Graphene-based sensors exhibit high sensitivity to mechanical deformations [8] and pressure changes while maintaining minimal power consumption—an essential feature for long-duration space missions. Furthermore, graphene demonstrates thermal stability and can endure extreme temperature fluctuations, making it a promising material for aerospace applications [9].

Despite its advantages, pristine graphene has limitations in terms of sensitivity for strain and pressure sensing. To enhance its performance, researchers have explored doping and functionalization techniques [10,11]. The incorporation of heteroatoms such as nitrogen, boron, and oxygen alters graphene’s electronic properties, improving its strain and pressure response. In addition, adding chemical functional groups or metal nanoparticles [12] to the surface of graphene can improve its sensitivity to external influences, such as pressure or strain. For example, hybrid structures containing graphene and metal nanoparticles can improve strain and pressure recognition. In addition, creating composite materials based on graphene and other polymers or materials can combine the advantages of graphene with the improved mechanical properties of other components [13].

In this study, we investigate graphene doped with six elements—boron, nitrogen, aluminum, silicon, sulfur, and gallium—chosen for their distinct valence states, atomic radii, and electronic characteristics. Elements from group IIIA (B, Al, Ga) act as p-type dopants and are of particular interest due to their ability to introduce holes and modify the local electronic environment [14,15,16,17]. Nitrogen is a well-established n-type dopant commonly used in graphene modification [18]. Silicon and sulfur, with their larger atomic radii and distinct bonding behavior, provide further contrast in both electronic interaction and lattice distortion [19,20]. This selection enables a systematic comparison of how different dopants influence the electronic response of graphene under biaxial strain.

To address the band gap problem, atoms of other elements are introduced into graphene to modify its electronic properties and promote the creation of a band gap [21], making it suitable for applications in electronics and sensors. As can be derived from data published by Singh A. and co-workers [8], their study found that doping graphene with elements like aluminum, boron, and nitrogen significantly enhances its strain sensitivity, with aluminum-doped graphene exhibiting the highest response. Another study [22] introduced a novel hybrid material combining graphene with graphitic carbon nitride (g-C_3_N_4_), which displayed enhanced piezoresistivity compared to pristine graphene, making it suitable for strain-sensing applications. Moreover, it has been reported [23] that the mechanical properties of graphene are highly sensitive to low doping concentrations; however, this sensitivity decreases when the dopant content exceeds a certain threshold. This observation is consistent with recent first-principles studies [24,25], which demonstrate that strain-induced redistribution of electronic states is strongly influenced by both the type of dopant and the applied mechanical strain. These findings provide a strategy for engineering the electronic properties of graphene to create strain-sensitive materials.

To further study the electronic and mechanical properties of such modified graphene nanomaterials, density functional theory (DFT) plays a special role and has become a powerful tool in materials science. DFT enables the theoretical study of electronic and mechanical properties under different strain and pressure conditions, allowing researchers to predict material behavior and optimize sensor performance. This theory was applied to analyze the electronic characteristics of materials, such as their conductivity, under mechanical stress. DFT is commonly used to determine the electronic characteristics of materials that influence their sensitivity to strain and pressure [26,27]. Moreover, previous studies have demonstrated that DFT modeling is not only useful at the atomic level but has also been extended to experimentally validated and mesoscale simulations of doped two-dimensional systems [28,29].

In this work, we present a comparative DFT-based investigation of graphene doped with six different atoms—B, N, Al, Si, S, and Ga—under biaxial strain ranging from −0.05 to +0.05. While most prior studies examine either individual dopants or strain effects in isolation, our approach combines both aspects in a consistent computational framework. This enables us to evaluate how different dopants influence strain-induced variations in the Fermi energy and the overall electronic structure, as reflected in the density of states. The findings contribute to understanding how doping and mechanical deformation can be used to tailor the electronic behavior of graphene-based materials.

## 2. Computational Details

All calculations were carried out using spin-unrestricted density functional theory (DFT), as implemented in the Dmol^3^ module [30]. It is widely recognized that simulations limited to the local density approximation (LDA) underestimate equilibrium distances and overestimate bond energy E_b_. Therefore, the generalized gradient approximation (GGA) was used to improve the accuracy of total and atomization energies, as well as energy barriers. The Perdew–Burke–Ernzerhof (PBE) functional, within the GGA scheme, was applied to describe exchange–correlation effects [31].

The DFT semi-core pseudopotential (DSPP) for core treatment with a double numerical basis set plus d-functions (DND) basis set was employed. The energy tolerance, maximum force, and displacement convergence were set at 2 × 10^−5^ Ha, 0.004 Ha/Å, and 0.005 Å, respectively. The Fermi–Dirac smearing function was applied with a smearing value of 0.005 Ha (1 Ha = 27.2114 eV) to improve the convergence of the self-consistent field (SCF) iterations. We used version 3.5 of the basis set library provided in the Dmol^3^ package for all calculations.

The real-space global cutoff radius was set to 4.5 Å. A 10 × 10 × 1 Monkhorst–Pack grid was set for the k-point meshes of the Brillouin zone sampling in this study. The self-consistent field (SCF) tolerance was set to 1 × 10^−6^ Ha. The direct inversion of the iterative subspace (DIIS) method was used to accelerate SCF convergence, with the DIIS subspace size set to 6.

The model system was a 5 × 5 graphene supercell containing 50 carbon atoms, without hydrogen termination at the edges. A 25 Å vacuum gap was added along the z-axis to eliminate artificial interactions between periodic layers.

## 3. Results and Discussion

This section presents an analysis of the electronic properties of graphene doped with boron, nitrogen, aluminum, silicon, sulfur, and gallium under symmetric biaxial strain. The calculations were performed using the density functional theory (DFT), with strain applied in both the x- and y-directions in the range of −0.05 to +0.05. The analysis emphasizes how the Fermi energy (E_F_) changes as a function of strain for each dopant.

### 3.1. Geometric and Structural Features of Doped Graphene

**Figure 1:**
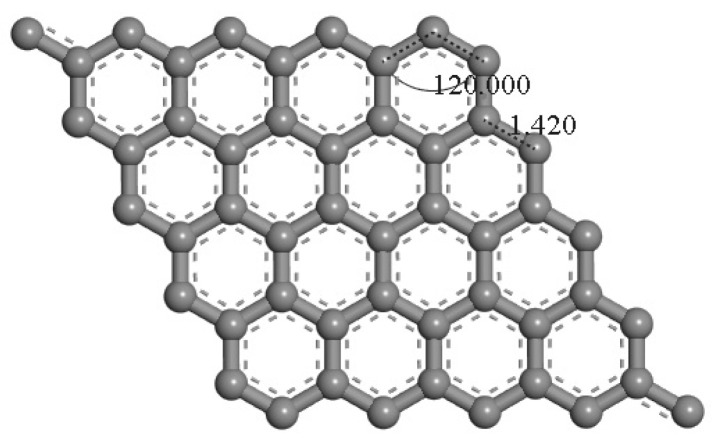
Top view of the optimized structure of pristine graphene.

**Figure 2:**
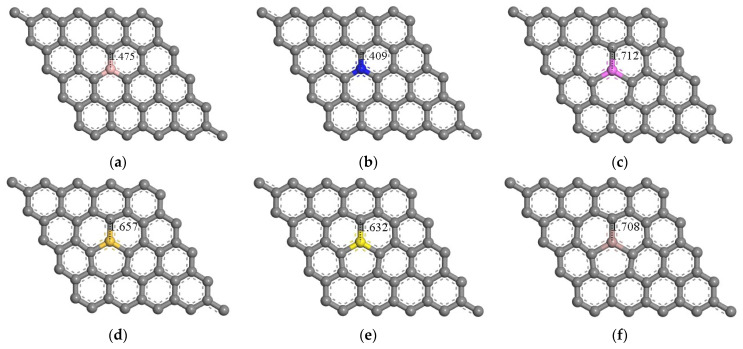
Relaxed geometries of 5 × 5 supercells of graphene doped with boron (**a**), nitrogen (**b**), aluminum (**c**), silicon (**d**), sulfur (**e**), and gallium (**f**) atoms. Gray atoms denote carbon atoms. Pink, blue, purple, yellow, dark yellow, and pale pink atoms denote boron, nitrogen, aluminum, sulfur, silicon, and gallium atoms. The bond lengths are in Å.

Figure 1 and Figure 2 show the optimized atomic structures of pristine graphene and graphene doped with boron, nitrogen, aluminum, silicon, sulfur, and gallium. In this study, monolayer graphene models were constructed using a 5 × 5 supercell (50 carbon atoms), where 1 carbon atom was substituted with a dopant atom, corresponding to a dopant concentration of 2%. This configuration was chosen to investigate how low-level substitutional doping affects the electronic properties of graphene, particularly in the context of potential sensing applications.

Geometry optimization was performed using DFT for each doped configuration. The optimized lattice parameters of the pristine graphene supercell were consistent with previously reported values [15]. For reference, the primitive unit cell of graphene contains two carbon atoms with a C–C bond length of 1.420 Å and 120° bond angles, forming the characteristic hexagonal lattice structure (Figure 1).

Upon doping, structural changes were observed in the local bonding environment around the dopant atoms. While B- and N-doped systems retained near-planar geometry with minimal deviation from the ideal C–C bond length, dopants such as Al, Si, S, and Ga introduced notable distortions. These include elongation of the C–X bond to 1.71–1.72 Å, depending on the dopant species. The C–X bond lengths are summarized in Table 1. The observed distortions are consistent with the larger atomic radii and distinct bonding characteristics of group IIIA and VIA elements compared to carbon. These structural modifications are expected to influence the electronic distribution, orbital hybridization, and local density of states, as further discussed in subsequent sections.

### 3.2. Strain-Induced Variation in Fermi Energy in Doped Graphene

**Figure 3:**
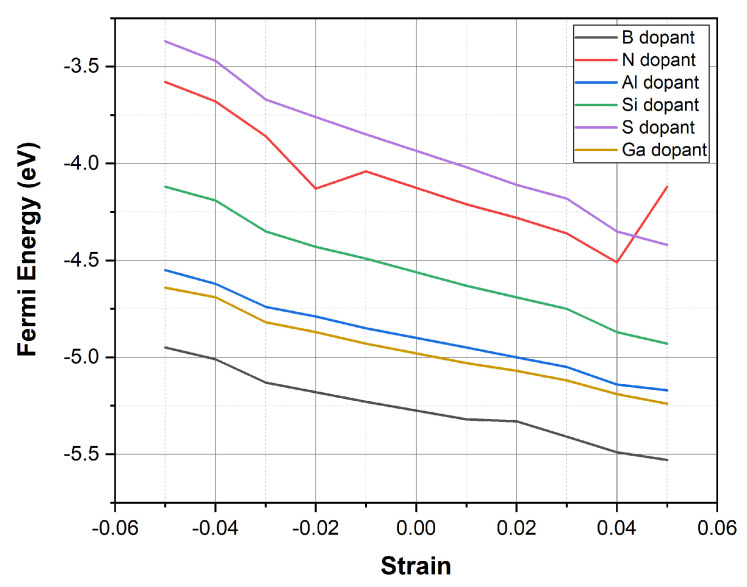
Fermi energy vs. biaxial strain for various dopants in graphene.

Table 2 and Table 3 present the computed Fermi energy (E_F_) values for each doped system under biaxial tensile strain (from +0.01 to +0.05) and compressive strain (from −0.01 to −0.05), which correspond to +1% to +5% and −1% to −5% changes in the lattice dimensions, respectively. These numerical results are also visualized in Figure 3, which shows the E_F_ shift as a function of applied biaxial strain for all dopants.

Among the studied dopants, graphene doped with gallium and aluminum shows the most significant variation in Fermi energy across the strain range. In Ga-doped graphene, E_F_ shifts from −4.64 eV at ε = −0.05 to −5.24 eV at ε = +0.05, corresponding to a total change of 0.60 eV. A similar trend is observed in Al-doped graphene, where E_F_ decreases from −4.55 eV to −5.17 eV (ΔE_F_ = 0.62 eV). These results suggest enhanced strain sensitivity in systems doped with group IIIA elements.

The pronounced decrease in Fermi energy observed under tensile strain is primarily attributed to a reduction in the π-orbital overlap as the C–C bond lengths increase. This structural elongation reduces orbital overlap and narrows the electronic bands, resulting in a downward shift in the Fermi level. Such effects are especially prominent in systems with heavier dopants, where lattice distortions are more significant.

The strong strain-induced shift in E_F_ observed for Ga- and Al-doped graphene highlights their potential for strain-sensitive applications. In particular, the consistent modulation of electronic structure under mechanical deformation suggests that such systems could be promising candidates for integration into graphene-based strain sensors.

In contrast, B- and N-doped graphene exhibit more modest shifts in E_F_, ranging from −4.95 eV to −5.53 eV and from −3.58 eV to −4.12 eV, respectively. Although the changes are less pronounced, the nearly linear variation with strain may be beneficial for applications requiring predictable and stable electronic responses.

S- and Si-doped systems exhibit intermediate behavior. The E_F_ in S-doped graphene shifts from −3.37 eV to −4.42 eV, while for Si-doping, it varies from −4.12 eV to −4.93 eV. These results suggest that although the strain-induced modulation is less extreme than in Ga- or Al-doped systems, sulfur and silicon still offer meaningful electronic tunability.

### 3.3. Density of States Analysis of Doped Graphene Under Strain

**Figure 4:**
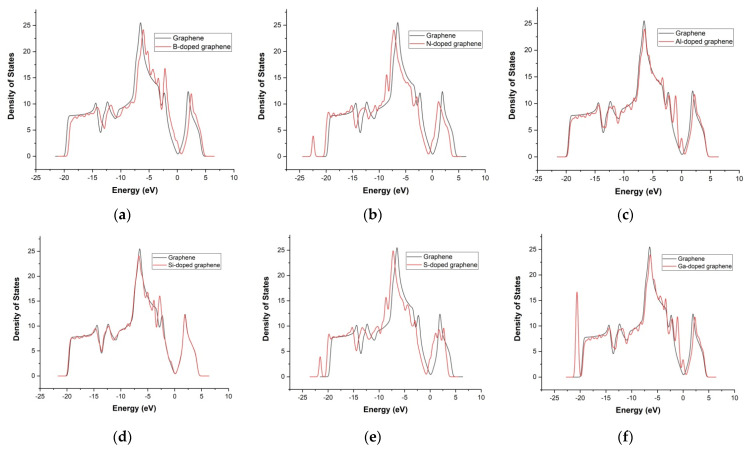
Density of electronic states of initial (pristine) graphene and doped graphene with boron (**a**), nitrogen (**b**), aluminum (**c**), silicon (**d**), sulfur (**e**), and gallium (**f**) atoms.

To further explore how doping and strain affect the electronic structure of graphene, we computed the total density of states (DOS) for pristine and doped systems under biaxial strain. This analysis provides insight into how different dopants modify the distribution of electronic states near the Fermi level (E_F_), which is critical for understanding their strain responsiveness and sensor potential. As shown in Figure 4, pristine graphene exhibits a characteristic linear DOS near the Dirac point, vanishing at the Fermi level. This behavior reflects its semi-metallic nature and explains its limited intrinsic sensitivity to electronic modulation under strain.

To investigate the modifications of the conduction and valence bands, an analysis of the DOS near the Fermi level was conducted for each doped configuration.

In the absence of strain, substitutional doping with boron introduces p-type behavior in graphene because it has one fewer valence electron than carbon. This causes a downward shift of the Fermi level (E_F_) below the Dirac point, as shown in Figure 4a. The DOS at E_F_ is no longer zero, unlike in pristine graphene. Previous studies have reported that boron doping in a 50-carbon-atom matrix can induce a band gap of approximately 0.14 eV, in agreement with our results [17,32].

Nitrogen, possessing one additional valence electron compared to carbon, acts as an n-type dopant when substituted into the graphene lattice. This causes an upward shift of the Fermi level above the Dirac point, leading to downward band bending in the electronic structure and an increase in electron concentration near the conduction band (Figure 4b). And the DOS shows notable increases at −23 eV and −8.5 eV, as well as near E_F_ [15]. Our calculations also indicate a band gap of ~0.14 eV, consistent with previously published data [17].

Although aluminum has more electrons than carbon, its larger atomic size and lower electronegativity cause E_F_ to shift downward (Figure 4c), suggesting p-type behavior. The incorporation of Al results in the introduction of hole-like carriers and noticeable distortion of the electronic structure [33].

Silicon, with four valence electrons, forms covalent bonds with adjacent carbon atoms, disrupting graphene’s symmetry and opening a band gap. The Si-doped structure exhibits semiconducting behavior with a calculated band gap of approximately 0.3 eV and no electronic states at E_F_ (Figure 4d), consistent with previous findings [34].

Sulfur-doped graphene shows an upward shift of the E_F_ into the conduction band (Figure 4e), indicating n-type conductivity and electron-donating behavior. Our calculated band gap for S-doped graphene is ~0.13 eV, which aligns with other reports [35]. Although this issue is controversial in research, other studies have found that sulfur plays an acceptor role in S-doped graphene, leading to p-type behavior [36,37].

Gallium doping leads to an upward shift in the Dirac point (Figure 4f) and a downward movement of E_F_ into the valence band, suggesting p-type behavior. The DOS reveals significant changes, particularly at −21 eV and −11 eV, as well as between −6 eV and −0.5 eV, where new states appear. The band gap in this configuration expands to approximately 0.35 eV, in agreement with earlier studies [38].

To examine the effect of mechanical deformation, biaxial strain in the range of −0.05 to +0.05 was applied to the graphene structures. The applied biaxial strain was defined as

(1) $$\varepsilon_{x}=\frac{a_{x}-a}{a},\;\varepsilon_{y}=\frac{b_{y}-b}{b}$$

where $a_{x}$ and $b_{y}$ are the lattice constants along the x(y) direction after stretching and compression, respectively.

**Figure 5:**
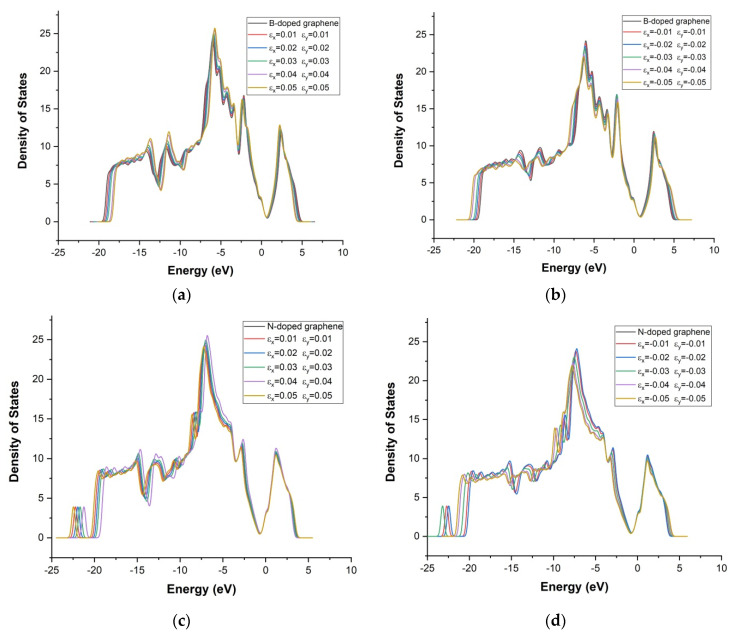

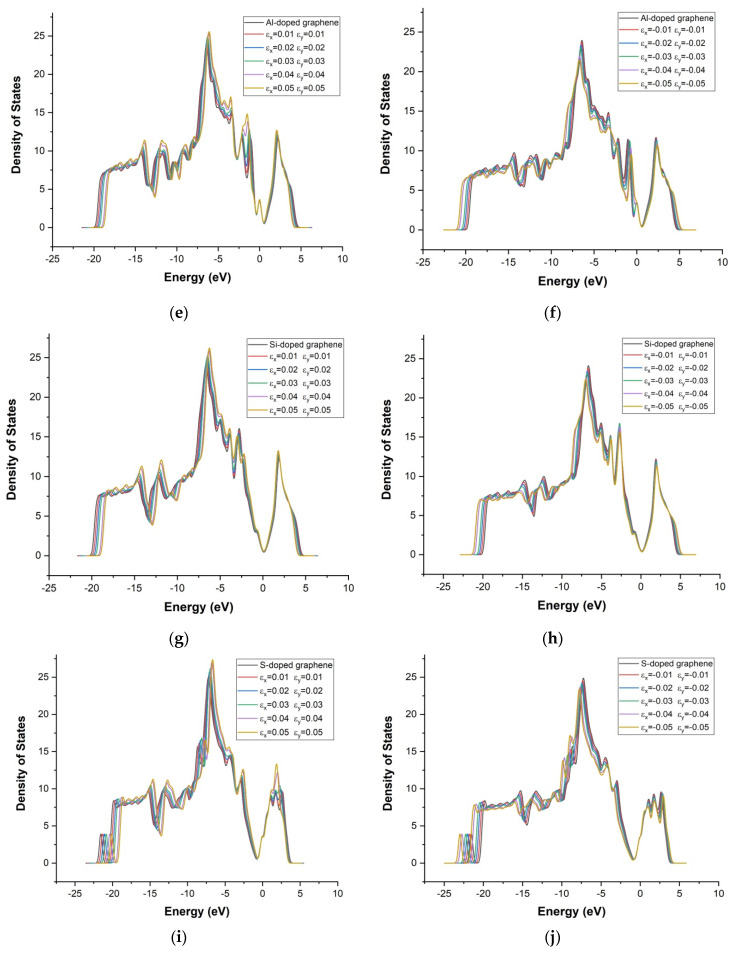

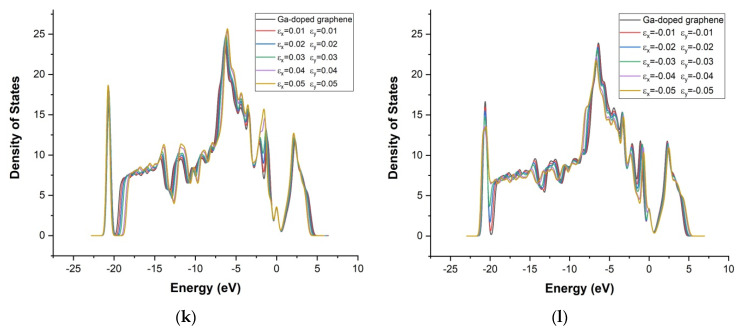
Density of states of graphene doped with boron (**a**,**b**), nitrogen (**c**,**d**), aluminum (**e**,**f**), silicon (**g**,**h**), sulfur (**i**,**j**), and gallium (**k**,**l**) after biaxial tensile and compressive deformation.

The effect of biaxial strain on graphene doped with boron, nitrogen, aluminum, silicon, sulfur, and gallium was investigated. In Figure 5, the density of states (DOS) plots under strain are shown. The DOS near the Fermi level is specifically examined to evaluate strain-induced changes in the conduction and valence bands.

The density of states (DOS) of boron-doped graphene under biaxial tensile and compressive strain is illustrated in Figure 5a,b. Under maximum tensile strain (ε_x_ = +0.05, ε_y_ = +0.05), the DOS shows pronounced peaks across the valence band region (from −18 eV to −3 eV), indicating an increased density of available electronic states. A distinct peak is also observed in the conduction band near +3 eV. These features suggest that significant tensile strain enhances the electronic activity of boron-doped graphene in comparison to the pristine graphene. Under compressive strain (ε_x_ = ε_y_ = −0.01 to −0.05), the DOS structure is modified differently. Enhanced peak intensities are observed, particularly at −14 eV for ε_x_ = −0.05, ε_y_ = −0.05 and at −2 eV for ε_x_ = −0.03, ε_y_ = −0.03. Overall, compression leads to a redistribution of states, with localized increases in both the valence and conduction bands, though less pronounced than under tension.

The DOS profiles under biaxial strain for nitrogen-doped graphene are shown in Figure 5c,d. Under tensile strain at (ε_x_ = +0.04, ε_y_ = +0.04), intensified peaks are observed at −18, −14.8, −12, −11.7, −9.8, −6.5, and −3 eV, along with the appearance of additional states near −1 eV in the conduction band. In contrast, reductions in density are seen at −14, −10.5, and −9.5 eV, indicating that a strain-induced redistribution of states near the Fermi level has occurred. Under compressive strain (ε_x_ = −0.02, ε_y_ = −0.02), the DOS (Figure 5d) is significantly enhanced at −15, −5.5, and −3 eV, as well as at +1 eV in the conduction band. These findings suggest that the electronic structure of N-doped graphene can be notably modified by even moderate levels of strain, particularly in the vicinity of E_F_.

The DOS of Al-doped graphene under biaxial strain is shown in Figure 5e,f. Under maximum tensile strain (ε_x_ = +0.05, ε_y_ = +0.05), increased DOS intensity is observed at −16, −15.2, −14, −11, −9, −7, −5.5, −3, and −1 eV, as well as above the Fermi level, including a distinct peak in the conduction band at +2.5 eV. A reduction in DOS is also noted at −13 eV, suggesting that a selective redistribution of states occurs under tension. Under compressive strain (ε_x_ = −0.05, ε_y_ = −0.05), pronounced peaks are exhibited at −14, −11, −10, −9.8, −7, −5.5, and −1 eV, with additional features in the conduction band at +3.0, +4.0, and +4.8 eV (Figure 5f). At lower compression (ε_x_ = −0.01, ε_y_ = −0.01), a general increase in peak intensity is also observed, though less prominently. These findings confirm that the electronic structure of aluminum-doped graphene is significantly modified under both tensile and compressive strain, particularly through the emergence of states above the Fermi level.

The effect of biaxial strain on the DOS of Si-doped graphene is illustrated in Figure 5g,h. Under maximum tensile strain (ε_x_ = +0.05, ε_y_ = +0.05), enhanced peaks are observed in the DOS at −17.5, −16, −15.5, −14.8, −12, −6, −5, −4.5, −3, and −2.5 eV, accompanied by a significant increase in the conduction band at +2.5 eV (Figure 5g). During compressive deformation, increased DOS intensities are noted at (ε_x_ = −0.01, ε_y_ = −0.01), particularly at −15, −13, −6, and −5 eV, with additional states detected at +2 eV in the conduction band (Figure 5h). At a higher compression level (ε_x_ = −0.03, ε_y_ = −0.03), an increase in the DOS is also seen at −3 eV in the valence band. These variations indicate that the DOS of Si-doped graphene is significantly influenced by both tensile and compressive strain near the Fermi level.

The DOS for S-doped graphene under biaxial strain is shown in Figure 5i,j. Under maximum tensile strain (ε_x_ = +0.05, ε_y_ = +0.05), enhanced DOS peaks are observed across a wide energy range, including −18, −16, −15.5, −14.8, −13, −10, −8, −5.5, −4.5, and −2 eV. Additional states are also detected above the Fermi level, with a pronounced peak appearing at +2.5 eV in the conduction band (Figure 5i). Under compressive strain (ε_x_ = −0.01, ε_y_ = −0.01), intensified DOS features are noted at −15.5, −6.5, −4.5, and −2.5 eV, accompanied by the emergence of new states above E_F_. In the conduction band, increases in DOS are seen at +1.5, +2.0, and +2.5 eV (Figure 5j), indicating enhanced electronic activity near the conduction edge. These observations suggest that the electronic structure of graphene is modified by sulfur doping in a strain-dependent manner.

The DOS profiles for Ga-doped graphene under biaxial strain are presented in Figure 5k,l. Under maximum tensile strain (ε_x_ = +0.05, ε_y_ = +0.05), increased intensity in the DOS is revealed at −16, −15.5, −14, −11, −8, −7, −5.5, −5, −4.8, −3.5, −1, and −0.5 eV, along with a prominent feature observed in the conduction band at +2.0 eV (Figure 5k). A significant enhancement of electronic states across both the valence and conduction bands is indicated by these results. Under compressive strain (ε_x_ = −0.01, ε_y_ = −0.01), several peaks are also intensified, although a reduction in DOS is noted at −20 and −13.5 eV, suggesting localized suppression of valence states (Figure 5l). Additionally, at (ε_x_ = −0.03, ε_y_ = −0.03), a clear increase in DOS is observed around −3 eV, indicating a further strain-dependent redistribution of electronic states.

To contextualize these findings and clarify the computational approach, the calculated Fermi energies and density of states depend on the choice of the exchange–correlation functional. The PBE functional, used in this study within the GGA framework, is known to underestimate absolute band gap values. However, as our analysis focuses on relative Fermi level shifts and qualitative electronic trends under strain, the use of PBE is appropriate for the scope of this work. No cross-validation with hybrid functionals was performed, as the goal was to compare strain-induced effects across doped systems rather than to obtain absolute band gap values.

### 3.4. Strain Sensitivity and Material Design Considerations

The observed correlation between applied biaxial strain and Fermi energy shift provides a fundamental design principle for developing piezoresistive materials based on doped graphene. Among all studied dopants, Ga- and Al-doped graphene exhibit the most pronounced and consistent shifts in E_F_ under relatively small biaxial strain (±0.05), underscoring their potential as highly sensitive elements in strain-responsive devices. These effects arise from the strong interaction between the dopants and the π-electron system of graphene, leading to notable redistributions of electronic states near the Fermi level.

The findings suggest that dopant selection is a critical parameter in tuning the electromechanical response of graphene and achieving desired sensor performance characteristics. Moreover, the results confirm that even low levels of symmetric biaxial strain is sufficient to modulate the electronic properties, provided the appropriate dopants are selected.

These findings support the rational design of functional materials for flexible electronics and strain-responsive sensors, particularly in aerospace structures and wearable technologies.

## 4. Conclusions

In this study, we employed density functional theory (DFT) to investigate the effect of biaxial mechanical strain on the electronic properties of graphene doped with B, N, Al, Si, S, and Ga. The Fermi energy served as the key indicator of strain sensitivity, evaluated across a symmetric deformation range from −0.05 to +0.05.

Our results demonstrate that the electronic response of doped graphene is strongly influenced by the dopant type. Among the studied elements, gallium and aluminum induced the most significant shifts in Fermi energy—up to 0.6 eV—highlighting their potential for high sensitivity and electronic tunability. In contrast, boron and nitrogen produced smaller yet more linear and stable changes, which may be advantageous for applications requiring consistent sensor performance.

These findings emphasize the critical role of dopant selection in engineering the strain responsiveness of graphene-based materials and identify Ga- and Al-doped systems as promising candidates for high-performance strain sensors. Future work may involve the experimental integration of these configurations into functional devices and an extension of the theoretical framework to larger strain magnitudes or more complex loading scenarios.

## Figures and Tables

**Table 1:** Calculated C–X bond lengths and structural interpretations for graphene doped with various atoms.

Dopant	C–X Bond Length (Å)	Deviation from C–C (1.42 Å)	Interpretation
B	1.47	+0.05	Slight elongation compared to pristine C–C bond
N	1.41	−0.01	Almost unchanged; close to C–C bond length
Al	1.72	+0.30	Significant elongation due to large atomic radius
Si	1.66	+0.24	Moderate elongation; size mismatch; and different bonding nature
S	1.63	+0.21	Similar elongation; electronegativity contrast; and out-of-plane distortion
Ga	1.71	+0.29	Comparable to Al; induces local lattice expansion

**Table 2:** Fermi energy values (eV) of doped graphene systems under biaxial tensile strain (ε = +0.01 to +0.05).

Strain	B	N	Al	Si	S	Ga
+0.05	−5.53	−4.12	−5.17	−4.93	−4.42	−5.24
+0.04	−5.49	−4.51	−5.14	−4.87	−4.35	−5.19
+0.03	−5.41	−4.36	−5.05	−4.75	−4.18	−5.12
+0.02	−5.33	−4.28	−5.00	−4.69	−4.11	−5.07
+0.01	−5.32	−4.21	−4.95	−4.63	−4.02	−5.03

**Table 3:** Fermi energy values (eV) of doped graphene systems under biaxial compressive strain (ε = −0.01 to −0.05).

Strain	B	N	Al	Si	S	Ga
−0.05	−4.95	−3.58	−4.55	−4.12	−3.37	−4.64
−0.04	−5.01	−3.68	−4.62	−4.19	−3.47	−4.69
−0.03	−5.13	−3.86	−4.74	−4.35	−3.67	−4.82
−0.02	−5.18	−4.13	−4.79	−4.43	−3.76	−4.87
−0.01	−5.23	−4.04	−4.85	−4.49	−3.85	−4.93

## Data Availability

The original contributions presented in this study are included in the article. Further inquiries can be directed to the corresponding author.
